# Refining clinical trial inclusion criteria to optimize the standardized response mean of the CMTPedS

**DOI:** 10.1002/acn3.51145

**Published:** 2020-08-06

**Authors:** Kayla M. D. Cornett, Manoj P. Menezes, Paula Bray, Rosemary R. Shy, Isabella Moroni, Emanuela Pagliano, Davide Pareyson, Tim Estilow, Sabrina W. Yum, Trupti Bhandari, Francesco Muntoni, Matilde Laura, Mary M. Reilly, Richard S. Finkel, Katy J. Eichinger, David N. Herrmann, Michael E. Shy, Joshua Burns, CMTPedS Study Group

**Affiliations:** ^1^ School of Health Sciences University of Sydney The Children’s Hospital at Westmead Sydney New South Wales Australia; ^2^ Paediatrics and Child Health University of Sydney The Children’s Hospital at Westmead Sydney New South Wales Australia; ^3^ Department of Pediatrics Carver College of Medicine University of Iowa Iowa City Iowa; ^4^ Fondazione IRCCS Istituto Neurologico Carlo Besta Milan Italy; ^5^ Department of Occupational Therapy The Children’s Hospital of Philadelphia Philadelphia Pennsylvania; ^6^ Division of Neurology Perelman School of Medicine The Children's Hospital of Philadelphia University of Pennsylvania Philadelphia Pennsylvania; ^7^ UCL Institute of Child Health and National Institute for Health Research Great Ormond Street Hospital Biomedical Research Centre London UK; ^8^ MRC Centre for Neuromuscular Diseases UCL Queen Square Institute of Neurology University College London London UK; ^9^ Translational Neurosciences (Pediatrics) St. Jude Children's Research Hospital Memphis Tennessee; ^10^ Department of Neurology University of Rochester Rochester New York; ^11^ Department of Neurology Carver College of Medicine University of Iowa Iowa City Iowa

## Abstract

The CMT Pediatric Scale (CMTPedS) is a reliable, valid, and responsive clinical outcome measure of disability in children with CMT. The aim of this study was to identify the most responsive patient subset(s), based on the standardized response mean (SRM), to optimize the CMTPedS as a primary outcome measure for upcoming clinical trials. Analysis was based on a 2‐year natural history data from 187 children aged 3–20 years with a range of CMT genetic subtypes. Subsets based on age (3–8 years), disability level (CMTPedS score 0–14), and CMT type (CMT1A) increased the SRM of the CMTPedS considerably. Refining the inclusion criteria in clinical trials to younger, mildly affected cases of CMT1A optimizes the responsiveness of the CMTPedS.

## Introduction

The CMT Pediatric Scale (CMTPedS) is a reliable, valid, and sensitive clinical outcome measure of disease severity in children and adolescents aged 3–20 years.^1^ Recent natural history data shows significant progression over 2 years at a rate of 2.4 ± 4.9 (14%) for all genetic subtypes of CMT (*P* < 0.001) and 1.8 ± 4.2 (12%) for CMT1A (*P* < 0.001).^2^ Although this rate of progression was significant, due to the slowly progressive nature and phenotypic variability^3^ of CMT there is value determining if there are subsets of patients that are more responsive on the CMTPedS to maximize power, and reduce sample size, in clinical trials by refining the inclusion criteria.

Responsiveness is the ability of an outcome measure to detect change over time.^4^ The standardized response mean (SRM) is an effect size index used to gauge the responsiveness of outcome measures, calculated by dividing the mean change by the standard deviation of the change. An SRM >0.8 is considered to indicate large responsiveness, 0.5–0.8 moderate, and 0.2–<0.5 low.^4^ Based on natural history data for all types of CMT,^2^ the SRM of the CMTPedS is 0.5 and for patients with CMT type 1A the SRM is 0.4. The aim of this study was to determine the most responsive patient subset(s), based on the SRM, to optimize the CMTPedS as a primary outcome measure for upcoming clinical trials.

## Methods

187 participants aged 3–20 years enrolled in the Inherited Neuropathy Consortium were assessed at baseline and after 2 years using the CMTPedS as previously described (Table 1).^2^ Baseline variables were iteratively correlated with CMTPedS change scores to determine the most responsive patient subsets. Baseline variables considered for optimization iterations were age, height, weight, gender, foot deformity, disability level, and CMT genetic subtype. Foot deformity was assessed using the Foot Posture Index.^5^ Disability was defined by a CMTPedS score of 0–14 (mild), 15–29 (moderate), and 30–44 (severe).^6^

**Table 1:** Participant characteristics.

Characteristic	Baseline	Follow‐up
Age, y	9.8 ± 3.9	11.8 ± 3.8
Height, m	1.40 ± 0.22	1.49 ± 0.20
Weight, kg	38.1 ± 16.4	45.2 ± 18.5
Foot posture index, −12 to 12	1.1 ± 4.3	0.5 ± 4.5
CMTPedS, 0 to 44	17.3 ± 9.1	19.6 ± 9.4

Values are mean ± SD.

### Statistical analysis

Data were analyzed using SPSS v. 25.0 (IBM Corp. Armonk, NY). All data were assessed for normality and the appropriate parametric or nonparametric test subsequently employed. Bivariate correlations were used to determine potential variables for the optimization. Disease progression and SRM were calculated for patient subgroups of significantly correlated variables. An alpha level of 0.05 was used for statistical significance.

## Results

Baseline age, height, weight and disability level were significantly correlated with the CMTPedS change score (*P* < 0.05). Due to high intercorrelations between age, height, and weight (*r* > 0.84, *P* < 0.001), only age was considered for optimization. The SRM of the CMTPedS increased considerably for several age and disability level subsets (Table 2). In particular, children aged 3–8 years with any type of CMT and a mild level of disability had a SRM of 0.8. For children aged 3–8 years with CMT1A and a mild level of disability, the SRM was 0.9. There were not enough children to evaluate responsiveness within other genetic subtypes such as CMT1B, CMT2A, CMT4C.

**Table 2:** CMTPedS responsiveness by baseline age, disability level, and CMT subtype.

Criterion	All CMT			CMT1A		
	Mean ± SD (n)	SRM	Sample size per arm^1^	Mean ± SD	SRM	Sample size per arm^1^
Whole sample	2.4 ± 4.9^2^ (*n* = 187)	0.5	66	1.8 ± 4.2^2^ (*n* = 111)	0.4	86
Mild disability	3.3 ± 5.0 (*n* = 79)	0.7	37	2.8 ± 4.3 (*n* = 58)	0.7	38
Moderate disability	2.0 ± 4.9 (*n* = 89)	0.4	95	0.7 ± 3.8 (*n* = 52)	0.2	463
Severe disability	0.5 ± 2.9 (*n* = 19)	0.2	529	−3.0 (*n* = 1)	N/A	N/A
Aged 11–20 years	1.7 ± 4.0 (*n* = 69)	0.4	87	1.2 ± 4.1 (*n* = 49)	0.3	184
Aged 3–10 years	2.9 ± 4.9 (*n* = 95)	0.6	45	2.2 ± 4.2 (*n* = 62)	0.5	58
Aged 3–9 years	3.3 ± 4.9 (*n* = 84)	0.7	35	2.5 ± 4.2 (*n* = 54)	0.6	45
Aged 3–8 years	3.5 ± 5.0 (*n* = 69)	0.7	33	2.7 ± 4.2 (*n* = 43)	0.6	38
Aged 3–10 years and mild disability	3.1 ± 4.6 (*n* = 53)	0.7	35	2.8 ± 4.2 (*n* = 44)	0.7	36
Aged 3–9 years and mild disability	3.3 ± 4.6 (*n* = 49)	0.7	31	3.0 ± 4.2 (*n* = 40)	0.7	31
Aged 3–8 years and mild disability	3.7 ± 4.5 (*n* = 42)	0.8	24	3.5 ± 3.9 (*n* = 33)	0.9	20

^1^ Sample size calculated for a 2‐year randomized (1:1) double‐blind, parallel‐group, placebo‐controlled trial of an intervention aiming to halt the rate of progression per treatment arm. SRM, standardized response mean.

## Discussion

Refining the inclusion criteria in clinical trials to younger, mildly affected cases of CMT1A would optimize the SRM of the well‐validated CMTPedS. As CMT is a progressive disease, intervening at the earliest stages of the disease will be important to halt or modify disease progression. The importance of early intervention has been shown in other progressive neuromuscular conditions such as Spinal Muscular Atrophy where intervening early and even presymptomatically has the best results.^7^, ^8^

Sample size considerations are important in rare diseases. By determining the most responsive patient subsets, based on the SRM of the CMTPedS, recruitment will be faster and trials will be more economical. For example, for a 2‐year randomized (1:1) double‐blind, parallel‐group, placebo‐controlled trial of an intervention aiming to halt the rate of progression would require 24 participants per arm for children aged 3–8 years with mild disability level compared to 66 per arm if inclusion criteria are not refined (Table 2). Note that adjustments would need to be made for correlation between pretest/posttest scores, loss to follow‐up, and nonadherence. In CMT1A, the required sample size per arm would be 20 for children aged 3–8 years with mild disability level, whereas 86 per arm would be required if all cases of CMT1A were included.

The CMTPedS is a fit for purpose outcome measure for clinical trials of disease‐modifying, rehabilitative and surgical interventions in children and adolescents with CMT. Refining the inclusion criteria to younger, mildly affected cases of CMT1A optimizes the responsiveness of this well‐validated clinical outcome measure.

## Conflict of Interest

The authors have no conflicts of interest to disclose.

## Author Contributions

KMDC and JB performed study concept and design. KMDC, MPM, RRS, IM, EP, DP, TE, SWY, TB, FM, ML, RSF, KJE, DNH, MES, and JB performed data acquisition and analysis. KMDC, MES, and JB drafted the manuscript and figures.

**CMTPedS Study Group:** Kayla M. D. Cornett, PhD^1^, Manoj P. Menezes, PhD^2^, Rosemary R. Shy, MD^3^, Daniela Calabrese^4^, Maria Foscan^4^, Silvia Genitrini^4^, Isabella Moroni, MD^4^, Emanuela Pagliano, MD^4^, Davide Pareyson, MD^4^, Timothy Estilow, OTR/L^5^, Sabrina W. Yum, MD^6^, Trupti Bhandari, PT^7^, Francesco Muntoni, MD, FRCPCH^7^, Matilde Laura, PhD^8^, Mary M. Reilly, MD, FRCP^8^, Richard S. Finkel, MD^9^, Kate J. Eichinger, DPT^10^, David N. Herrmann, MBBCh^10^, Paula Bray, PhD^1^, Michael E. Shy, MD^11^ and Joshua Burns, PhD^1^.
